# Supplementary material for: Do All Critically Ill Patients with COVID-19 Disease Benefit from Adding Tocilizumab to Glucocorticoids? A Retrospective Cohort Study
Source: Viruses. 2023 Jan 20;15(2):294. doi: 10.3390/v15020294 (PMC9967307; doi:10.3390/v15020294)
Supplement: Supplementary file 1 [file viruses-15-00294-s001.zip › viruses-2111985-supplementary.pdf]

Supplementary Table S1. Therapies used by treatment group

	Treatment strategy			
	Glucocorticoids	Glucocorticoids + tocilizumab	p-value*	Total
	N= 597	N= 395		N= 992
Heparin, n(%)				
Prophylaxis	360 (60.3%)	223 (56.5%)		583 (58.8%)
Intermediate	173 (29.0%)	160 (40.5%)	<.001	333 (33.6%)
Full dose	14 (2.3%)	15 (3.8%)	0.184	29 (2.9%)
Remdesivir, n(%)	5 (0.8%)	12 (3.0%)	0.009	17 (1.7%)
Glucocorticoids, n(%)				
Standard dose (Dexamethasone, 6 mg/day)	565 (94.6%)	337 (85.3%)	<.001	902 (90.9%)
High dose (Methylprednisolone 2 mg/kg body weight/day)	32 (5.4%)	58 (14.7%)	<.001	90 (9.1%)
Tocilizumab, n(%)	0 (0.0%)	395 (100.0%)	<.001	395 (39.8%)
Anakinra, n(%)	0 (0.0%)	0 (0.0%)		0 (0.0%)
Delay in starting intervention, days				
Median (IQR)	0 (0.0, 2.0)	2 (1.0, 3.0)		1 (0.0, 3.0)
Started <=2 days after admission, n(%)	161 (27.0%)	210 (53.2%)	<.001	371 (37.4%)

*Chi-square test

Supplementary Table S2. Outcomes by treatment group

Outcomes	Treatment strategy			
	Glucocorticoids	Glucocorticoids + tocilizumab	p-value*	Total
	N= 597	N= 395		N= 992
HFNO, n(%)	69 (11.6%)	202 (51.1%)	<.001	271 (27.3%)
NIV, n(%)	38 (6.4%)	112 (28.4%)	<.001	150 (15.1%)
Invasive mechanical ventilation (IMV), n(%)	34 (5.7%)	52 (13.2%)	<.001	86 (8.7%)
Days between hospital admission and NIV, Median (IQR)	1.0 (0.0, 7.0)	2.0 (1.0, 4.5)	0.187	2.0 (1.0, 5.0)
Days between disease onset and IMV, Median (IQR)	7.0 (4.0, 15.0)	12.0 (8.0, 17.0)	0.028	11.0 (7.0, 16.0)
Days free from HFNO (days), Median (IQR)	24.0 (20.0, 27.0)	24.0 (20.0, 26.0)	0.683	24.0 (20.0, 26.0)
Days free from NIV (days), Median (IQR)	26.0 (23.0, 27.0)	25.0 (23.0, 27.0)	0.459	25.0 (23.0, 27.0)
Days free from IMV (days), Median (IQR)	23.0 (17.5, 25.5)	21.0 (15.0, 24.0)	0.287	21.0 (16.0, 25.0)
Days free from hospital (days), Median (IQR)	20.0 (14.0, 23.0)	14.0 (3.0, 18.0)	<.001	17.0 (10.0, 22.0)
Days free from ICU (days), Median (IQR)	23.0 (21.0, 26.0)	24.0 (20.0, 25.0)	0.551	24.0 (20.0, 26.0)
Death, n(%)	113 (18.9%)	79 (20.0%)	0.676	192 (19.4%)
Death in those requiring HFNO, n(%)	19 (27.5%)	55 (27.2%)	0.960	74 (27.3%)
Death in those requiring NIV, n(%)	13 (34.2%)	38 (33.9%)	0.975	51 (34.0%)
Death in those requiring IMV, n(%)	17 (50.0%)	25 (48.1%)	0.862	42 (48.8%)
Discharged before day 28, n(%)	440 (73.7%)	247 (62.5%)	<.001	687 (69.3%)
Discharged to home, n(%)	320 (72.7%)	190 (76.9%)	0.228	510 (74.2%)
Discharged to RSA, n(%)	120 (27.3%)	57 (23.1%)		177 (25.8%)
Main delays				
Days from symptoms onset to hospitalisation, median(IQR)	6 (3, 9)	6 (3, 8)	0.401	6 (3, 9)
Days from symptoms onset to ICU, median(IQR)	7 (5, 11)	9 (7, 12)	0.028	8 (6, 12)
Days from hospitalisation to ICU, median(IQR)	0 (0, 2)	3 (1, 6)	<.001	1 (0, 4)
Hospitalised in 2021, n(%)	166 (27.8%)	103 (26.1%)	0.549	269 (27.1%)

*Chi-square or Mann-Whitney test

Supplementary Table S3. Unadjusted and adjusted HR of death from fitting Cox regression models - after removing those starting high level glucocorticoids

	Hazard ratios of death (95% CI)	p-value
**Unadjusted**		
**Glucocorticoids**	1	
**Glucocorticoids + tocilizumab**	0.67 (0.46, 0.99)	0.042
**Adjusted for time-fixed covariates[1]**		
**Glucocorticoids**	1	
**Glucocorticoids + tocilizumab**	0.56 (0.35, 0.90)	0.016
**Adjusted for time-varying covariates[2]**		
**Glucocorticoids**	1	
**Glucocorticoids + tocilizumab**	0.66 (0.41, 1.07)	0.090
**Weighted[3]**		
**Glucocorticoids**	1	
**Glucocorticoids + tocilizumab**	0.59 (0.38, 0.93)	0.022

[1]standard Cox model adjusted forage, ethnicity, CCI, baseline CRP and PaO2-FiO2 ratio

[2]standard Cox model adjusted forage, ethnicity, CCI, baseline PaO2-FiO2 ratio and CRP and time-varying useof remdesivir invasive mechanical ventilation, PaO2-FiO2 ratio and CRP

[3]weighted Cox model controlled forage, ethnicity, CCI, baseline PaO2-FiO2 ratio and CRP and time-varyinguse of remdesivir, invasive mechanical ventilation, PaO2-FiO2 ratio and CRPusing IPW

Supplementary Table S4. Unadjusted and adjusted HR of death from fitting Cox regression models - after removing those aged 75+ or with solid cancer

	Hazard ratios of death (95% CI)	p-value
**Unadjusted**		
**Glucocorticoids**	1	
**Glucocorticoids + tocilizumab**	0.74 (0.48, 1.14)	0.169
**Adjusted for time-fixed covariates**[1]		
**Glucocorticoids**	1	
**Glucocorticoids + tocilizumab**	0.60 (0.35, 1.01)	0.057
**Adjusted for time-varying covariates**[2]		
**Glucocorticoids**	1	
**Glucocorticoids + tocilizumab**	0.77 (0.45, 1.32)	0.341
**Weighted**[3]		
**Glucocorticoids**	1	
**Glucocorticoids + tocilizumab**	0.64 (0.38, 1.07)	0.087

[1]standard Cox model adjusted forage, ethnicity, CCI, baseline CRP and PaO2-FiO2 ratio

[2]standard Cox model adjusted forage, ethnicity, CCI, baseline PaO2-FiO2 ratio and CRP and time-varying useof remdesivir invasive mechanical ventilation, PaO2-FiO2 ratio and CRP

[3]weighted Cox model controlled forage, ethnicity, CCI, baseline PaO2-FiO2 ratio and CRP and time-varyinguse of remdesivir, invasive mechanical ventilation, PaO2-FiO2 ratio and CRPusing IPW

Supplementary Table S5. Results from fitting the Cox regression model after restricting to participants who had not received a full vaccination cycle prior to hospital admission.

	Hazard ratios of death (95% CI)	p-value
**Unadjusted**		
Glucocorticoids	1	
Glucocorticoids + tocilizumab	0.84 (0.59, 1.22)	0.362
**Adjusted for time-fixed covariates[1]**		
Glucocorticoids	1	
Glucocorticoids + tocilizumab	0.59 (0.37, 0.93)	0.024
**Adjusted for time-varying covariates[2]**		
Glucocorticoids	1	
Glucocorticoids + tocilizumab	0.61 (0.37, 0.98)	0.042
**Weighted[3]**		
Glucocorticoids	1	
Glucocorticoids + tocilizumab	0.64 (0.42, 0.98)	0.038

[1]standard Cox model adjusted forage, ethnicity, CCI, baseline PaO2-FiO2 ratio and CRP

[2]standard Cox model adjusted forage, ethnicity, CCI, baseline PaO2-FiO2 ratio and CRP and time-varying useof remdesivir invasive mechanical ventilation, PaO2-FiO2 ratio and CRP

[3]weighted Cox model controlled forage, ethnicity, CCI, baseline PaO2-FiO2 ratio and CRP and time-varyinguse of remdesivir, invasive mechanical ventilation, PaO2-FiO2 ratio and CRPusing IPW

Supplementary Table S6. Causes of deaths by treatment strategy

	Treatment strategy			
**Causes of death, n(%)**	**Glucocorticoids**	**Glucocorticoids + tocilizumab**	**p-value***	**Total**
MI or other cardiovascular	8 (7.1%)	7 (8.9%)		15 (7.8%)
Cardiac decompensation	16 (14.2%)	14 (17.7%)		30 (15.6%)
Infection	33 (29.2%)	23 (29.1%)		56 (29.2%)
Respiratory insufficiency	25 (22.1%)	30 (38.0%)		55 (28.7%)
Other concomitant morbidity	29 (25.7%)	5 (6.3%)		34 (17.7%)
Unknown	2 (1.8%)	0 (0.0%)		2 (1.0%)
Total	79 (100.0%)	113 (100.0%)		192 (100.0%)

*Chi-square

Supplementary Table S7. Incidence of adverse events by treatment group

Adverse event	Glucocorticoids	Glucocorticoids + tocilizumab	p-value	Total
Pulmonary Embolism	22/589 (3.7%)	24/391 (6.1%)	0.08&	46/980 (4.7%)
Sepsis	47/554 (8.5%)	34/81 (8.8%)	0.87&	81/941 (8.7%)
Neutropenia	10/432 (2.3%)	17/295 (5.8%)	0.02&	27/727 (3.7%)
Severe liver disease	44/429 (10.3%)	53/295 (18.0%)	0.003&	97/724 (13.4%)
Thrombosis	2/589 (0.3%)	5/391 (1.3%)	0.12*	7/980 (0.7%)

&Chi-square test

*Fisher exact test
